# The impact of prior obesity surgery on glucose metabolism after body contouring surgery: A pilot study

**DOI:** 10.17305/bb.2023.8827

**Published:** 2023-10-01

**Authors:** Saif Badran, Suhail A Doi, Atalla Hammouda, Hoda Khoogaly, Mohammad Muneer, Meis J Alkasem, Abdul-Badi Abou-Samra, Abdella M Habib

**Affiliations:** 1 Department of Population Medicine, College of Medicine, QU Health, Qatar University, Doha, Qatar; 2 Qatar Metabolic Institute, Hamad Medical Corporation, Doha, Qatar; 3 Department of Medicine, Weill Cornell Medicine Qatar, Qatar Foundation, Doha, Qatar; 4 College of Medicine, QU Health, Qatar University, Doha, Qatar

**Keywords:** Obesity, obesity surgery, bariatric surgery, body contouring surgery, surgical fat removal, insulin resistance, glucose homeostasis, metabolism

## Abstract

Body contouring surgery enhances physical appearance by means of surgical subcutaneous fat removal (SSFR). However, it remains unclear how SSFR may affect glucose metabolism and its broader effects on the endocrine system, especially in individuals who have undergone obesity (bariatric) surgery. This study aimed to evaluate the impact of SSFR on glucose excursion and insulin resistance in such patients, by examining them over three visits (within 1 week before surgery, 1 week after surgery, and 6 weeks after surgery). The independent impact of SSFR and history of obesity surgery on glucose homeostasis was evaluated in 29 participants, of whom ten patients (34%) had a history of obesity surgery. Indices of glucose metabolism were evaluated using cluster robust-error logistic regression. Results indicated that SSFR led to a gross improvement in insulin resistance at 6 weeks after the surgery in all patient’s irrespective of BMI, type 2 diabetes mellitus (T2D) status, or history of obesity surgery (OR 0.22; *p* ═ 0.042). However, no effect was observed on glucose excursion except for a transient increase at visit 2 (1 week after surgery) in those without prior obesity surgery. Interestingly, participants with a history of obesity surgery had approximately half the odds being in the upper tertile for HOMA-IR (OR 0.44; *p* ═ 0.142) and ten-folds lower odds of having severely abnormal glucose excursion (OR 0.09; *p* ═ 0.031), irrespective of their BMI, T2D status, or time post SSFR. In conclusion, this study showed that body contouring surgery through SSFR resulted in (at least) short-term improvement in insulin resistance (independent of the participant’s BMI, T2D status, or history of obesity surgery) without affecting glucose excursion under the GTT. On the contrary, obesity surgery may have a long-term effect on glucose excursion, possibly due to sustained improvement of pancreatic **β**-cell function.

## Introduction

Currently, there is a significant drift toward people seeking body contouring surgical interventions, such as dermo-lipectomy and liposuction, to quickly improve body appearance. This increase in demand can be attributed to several factors, including sedentary lifestyle, consumption of high energy diets, media emphasis on fitness and health, as well as the current paucity of effective and safe pharmacological treatment for overweight and obesity [1]. An additional push behind these body contouring surgeries is the recent advancement in safety and popularity of obesity surgeries, such as Roux-en-Y gastric bypass and sleeve gastrectomy, both of which are currently the most effective surgical interventions for treating obesity and type 2 diabetes (T2D). However, these procedures usually followed by a subsequent surgical intervention to remove excess residual subcutaneous fat and redundant skin to improve physical appearance [2, 3]. Surgical subcutaneous fat removal (SSFR), a main consequence of body contouring surgery, differs from other modalities of reducing body fat (such as diet, exercise, and obesity surgeries) since SSFR results in a sudden loss of adipocytes from the abdominal subcutaneous fat (ASF) compartment. On the other hand, other forms of fat reduction all result in a gradual decrease of both subcutaneous and intraabdominal adipocytes in terms of both size and quantity [4]. The metabolic impacts of the large volume subcutaneous fat removal during body contouring surgery are not known fully [5–7]. Several studies have investigated the latter, using different tests that assess glucose homeostasis, such as the homeostasis model assessment-estimated insulin resistance (HOMA-IR) [8–10] and fasting insulin levels [11, 12]. Fewer studies have assessed both fasting and postprandial glucose homeostasis using the insulin tolerance test [13], oral glucose tolerance test (OGTT) [14, 15], or the gold standard glucose clamp test [16, 17]. The existing studies have been summarized in one systematic review and five meta-analyses [6, 7, 18–21], and these syntheses suggest a possible improvement in insulin sensitivity, but a major challenge in interpreting these results is that they did not account for the heterogeneity of patients in terms of baseline body mass index (BMI), T2D status, and prior obesity (bariatric) surgery. This is of high importance to delineate the independent effect of SSFR on glucose homeostasis. To evaluate the latter, we decided to assess the independent impact of SSFR on both glucose excursion (which is the sum of pancreatic β cell function and insulin resistance) and insulin resistance (HOMA indices) while accounting for preoperative BMI, T2D status, and prior bariatric surgery history.

## Materials and methods

### Subjects

We studied 29 consecutive eligible patients who were planned to undergo SSFR that included either abdominoplasty or lower body lift surgery (liposuction cases were excluded) at Hamad General Hospital, in the period between July 2021 and December 2022. All subjects had a stable weight for at least six months before the surgery with a fluctuation of less than 3% of body weight. Patients with comorbidities were excluded except for T2D. Diabetic patients on insulin therapy were excluded. Patients with a history of obesity surgery were excluded if the surgery was less than two years before the body contouring surgery.

### Study design and reporting

The research design in this study was a quasi-experiment with three time points. A quasi-experimental design lacks individual patient randomization, but it has allocation of treatment by the researcher, and the longitudinal nature of this design means that the same patients act as their own control. This design was chosen because the classical experimental design (randomized controlled trial) is not appropriate for this type of study. Outcome variables of interest were measured at three time points which were the patient hospital visits (visit one: within 1 week before surgery, visit two: 1 week after surgery, and visit three: 6 weeks after surgery). The TREND reporting guideline for nonrandomized/quasi-experimental study designs was used to guide the reporting in this paper (Figure S1) [22].

### Patient measurements

Collected outcome variables during the three visits included patient age, gender, comorbidities and medications, history of obesity surgery, vital signs, body fat composition measurements using bioelectrical impedance analysis (TANITA^^®^^ segmental body composition scale) before and after surgery, details of the surgical procedure, including type of surgery and the weight of fat mass removed (in grams), OGTT using 75-gm oral glucose with six time points of glucose measurements (fasting (gtt0), 15 min (gtt15), 30 min (gtt30), 45 min (gtt45), 60 min (gtt60), and 120 min (gtt120) in mmol/L), fasting insulin (pmol/L) and C-peptide (nmol/L), hemoglobin A1c [HBA1c; (%)], lipid profile (LDL, HDL, and triglyceride in mmol/L), C-reactive protein [CRP; (mg/L)], interleukin-6 [IL-6; (pg/mL)], vitamin D (ng/mL). The HOMA-IR (anchored at 1 for normal insulin sensitivity) was calculated by means of the fasting plasma glucose and fasting C-peptide using the University of Oxford HOMA2 calculator [23]. For each of the GTT’s, glucose excursion was computed using Doi’s weighted average glucose (dwAG) [24, 25] and was categorized into four categories: dwAG0 ≤ 6.8, dwAG1 > 6.8 and ≤ 7.5, dwAG2 > 7.5 and ≤ 8.6, and dwAG3 > 8.6 mmol/L based on four levels of risk previously defined for women with gestational diabetes [24]. The four levels of dwAG reflect normal, impaired, abnormal, and severely abnormal dwAG, respectively. The dwAG has been validated [25] against the area under the GTT curve.

### Blood samples and assays

Fasting blood samples were collected, immediately processed, and stored frozen at −80 ^∘^C pending analysis. All assays were performed at the central laboratory of Hamad Medical Corporation, a laboratory accredited by the College of American Pathologist (CAP) and Joint Commission International (JCI).

Plasma glucose was measured using a hexokinase-based enzymatic method, the coefficient of variation for the assay was 1.2% at a mean glucose value of 5.3 mmol/L during the study period. Total cholesterol, triglycerides, and high-density lipoprotein cholesterol (HDL-C) levels were measured enzymatically. Low-density lipoprotein cholesterol (LDL-C) was calculated using the Friedewald equation. Serum 25(OH)D concentration (included both vitamin D2 and vitamin D3 fractions) was measured using electrochemiluminescence immunoassay (Vitamin D Total II, Roche, North America, USA). Plasma insulin and C-peptide concentrations were measured on EDTA plasma (0.1 mL) using a sandwich-based assay on microparticles detected by fluorescence according to the manufacturer recommendations (insulin and C-peptide Elecsys kits, Roche, North America, USA). The detection ranges were between 0.2–1000 mIU/mL and 0.01–40 ng/mL, for insulin and C-peptide, respectively. The intra-assay and inter-assay variations were less than 5% for both assays. The plasma concentration of CRP was measured using a particle-enhanced immunoturbidimetric assay following the manufacturer recommendation (cobas CRP Test, Roche Diagnostics, North America, USA); the CRP in the diluted plasma binds with the CRP antibody on latex particles; the concentration of CRP is calculated as a function of the changed absorbance measured at 525 nm and 625 nm which is in relation to the amount of agglutination. The detection range is 3.0–400 mg/L and intra- and inter-assay variations are less than 4%. IL-6 was measured by a non-competitive (sandwich) chemiluminescent immunoassay (Elecsys^®^ IL-6, Roche Diagnostic, North America USA). The assay measures a range of 1.5-5000 pg/mL, with an inter-assay precision of 17.4% (at 1.82 pg/mL) and 2.0% (at 4461 pg/mL) and a stated reference value <7 pg/mL.

All subjects had an OGTT with a 75-g glucose challenge and blood sampling at 0, 15, 30, 60, 90, and 120 min. Blood samples during the OGTT were collected in plain microtubes, rapidly centrifuged in a micro-centrifuge, and the supernatant serum was assayed for glucose concentrations using Analox (Analox Instrument Ltd, GM9, UK).

### Ethical statement

This study was approved by the Institutional Review Board at Hamad Medical Corporation and Qatar University (MRC-01-20-466 and QU-IRB 1412-EA/20, respectively), and by the Institutional Bio-safety Committee at Qatar University (QU-IBC-2020/066). All subjects signed an informed consent before starting the study.

### Statistical analysis

Descriptive statistics were computed (median and interquartile range or number and percent) to report patient variables across time points. Because the data collected over time (three time points) are correlated, the methods used for longitudinal data analysis accounted for the correlated nature of the data. A cluster robust error logistic regression analysis was conducted to assess predictors of glucose excursion with the clusters being the individual patient. Two outcomes were analyzed in two separate analyses, with outcomes being either upper tertile HOMA-IR (model 1) or severely abnormal (dwAG3) glucose excursion (model 2). Only patient characteristics deemed important prognostically for these outcomes were adjusted for in these models. The mass of fat removed was not included in the models because it was correlated with the degree of obesity and thus a proxy for it. Predictive margins from the logistic model were computed as a way of presenting model results in the scale of interest (probability), not in the estimation scale (logit) as the latter is more informative than odds ratios (OR). A predictive margin is a generalization of an adjusted mean applied to the nonlinear model (logistic regression model) thus using the estimated model to make predictions on different values of a covariate to evaluate its effect on the outcome. Stata version 15 (College Station, TX, USA) was used for all analyses and exact *P* values were reported throughout.

## Results

### Patients studied

The study included 29 patients (22 females and 7 males), all patients had at least one postoperative visit (15 patients completed both second and third visit, 7 patients completed the second visit only, and 7 patients completed the third visit only). Ten patients (37%) had a history of obesity surgery (six sleeve gastrectomy, two bypass surgery, two sleeve plus bypass surgery). Eleven patients (38%) were either lean or overweight, and the remaining 18 patients (62%) were obese. Five patients (17%) had T2D on oral medications, and none were on insulin therapy. A detailed medical history and complete physical examination revealed no other serious comorbidities or organ dysfunction in any participant. Average ASF removed during surgery was 2400 (range 1300–3600) g. Preoperatively, the median dwAG value was 7.0 mmol/L (interquartile range [IQR] 6.4–8.3), and median HOMA-IR was 1.6 (IQR 1.3–2.1). The Tanita full body composition analysis, complete lipid profile, and basic laboratory results are reported in Table 1. While the mean fat% and fat mass remain unchanged, on average, across visits, in a paired-difference linear regression analysis we find that for every percent difference in fat% the excised tissue in body contouring surgeries increased by 206.1 g (95% CI 26.1 g, 386.1 g).

**Table 1:** Characteristics of the study population

**Factor**	**Level**	**Visit 1**	**Visit 2**	**Visit 3**
Number of participants		29	22	22
Age (years)		43.0 (38.0, 50.0)	41.0 (37.0, 50.0)	43.0 (38.0, 51.0)
Sex	Male	7 (24.1%)	6 (27.3%)	5 (22.7%)
	Female	22 (75.9%)	16 (72.7%)	17 (77.3%)
Diabetic status	No	24 (83%)	20 (91%)	18 (82%)
	Yes	5 (17%)	2 (9%)	4 (18%)
dwAG value (mmol/L)		7.0 (6.4, 8.3)	6.9 (6.4, 8.7)	7.1 (5.7, 8.2)
AUC-glucose (mmol/L/2h)		16.6 (13.5, 20.4)	15.8 (14.4, 19.0)	15.4 (12.9, 18.2)
HOMA-IR		1.6 (1.3, 2.1)	1.7 (1.3, 2.0)	1.5 (1.2, 1.7)
History of bariatric surgery	Yes	10 (34%)	9 (41%)	7 (32%)
	No	19 (66%)	13 (59%)	15 (68%)
BMI category	< 30 kg/m^2^	11 (38%)	9 (41%)	7 (32%)
	≥ 30 kg/m^2^	18 (62%)	13 (59%)	15 (68%)
BMI (kg/m^2^)		31.7 (29.1, 33.6)	31.7 (29.1, 34.2)	32.0 (29.3, 34.2)
Bioelectrical impedance measures				
Body fat percent (%)		37 (33.6, 42.2)	37 (32.9, 42.9)	38.9 (34.1, 44.0)
Fat mass (kg)		32.4 (26.6, 37.4)	32.1 (26.6, 40.3)	32.5 (26.9, 37.4)
Total body water percent		44.4 (41.4, 47.1)	44.4 (41.4, 47.2)	43.8 (40.8, 46.1)
Basal metabolic rate (kJ/day)		5933 (5644, 6556)	5897.5 (5523, 6556)	6070.5 (5653, 7130)
Visceral fat rating		9 (6, 11)	8.5 (6, 12)	9 (6, 12)
Routine metabolic profile				
HbA1c (%)		5.4 (5.2, 5.6)	5.4 (5.2, 5.6)	5.3 (5.2, 5.6)
CRP (mg/L)		1 (1, 2.8)	1 (1, 2.9)	1 (1, 2.8)
IL-6 (pg/mL)		3 (1, 5)	3.5 (2, 5)	3 (1, 4)
Vitamin D (ng/mL)		26 (19, 36)	26.5 (21, 40)	25 (18, 40)
Cholesterol (mmol/L)		4.3 (3.8, 4.9)	4.3 (3.8, 4.8)	4.4 (3.7, 4.8)
Triglyceride (mmol/L)		0.8 (0.6, 1)	0.9 (0.6, 1)	0.8 (0.6, 1)
HDL (mmol/L)		1.3 (1.1, 1.7)	1.3 (1.1, 1.6)	1.2 (1.1, 1.7)
LDL (mmol/L)		2.8 (1.9, 3.4)	2.8 (1.9, 3.4)	2.8 (1.9, 3.3)

Data are presented as n (%) or median (IQR). dwAG: Doi's weighted average glucose; HOMA-IR: Homeostasis model assessment-estimated insulin resistance; BMI: Body mass index; HbA1c: Hemoglobin A1c; CRP: C-reactive protein; IL-6: Interleukin-6; HDL: High-density lipoprotein; LDL: Low-density lipoprotein; AUC: Area under the curve.

### Model 1 (HOMA-IR): Predictors of insulin resistance

The risk of severe insulin resistance (defined as having an upper tertile HOMA-IR level) was assessed in relation to SSFR, history of bariatric surgery, T2D status, and baseline BMI independently (Table 2). The median for HOMA-IR in the upper tertile (across all time points) was 2.18 (IQR 1.96–3.30).

**Table 2:** Predictors of insulin resistance (model 1: HOMA-IR) or abnormal glucose excursion (model 2: dwAG3)

**Variable**	Model 1 (HOMA-IR) ** OR (95% CI)	***p* values**	Model 2 (dwAG3) ** OR (95% CI)	***p* values**
*Time post SSFR*				
1 week after surgery*	1.30 (0.36, 4.67)	0.688	2.20 (0.56, 8.56)	0.256
6 weeks after surgery*	0.22 (0.05, 0.95)	0.042	1.05 (0.17, 6.34)	0.956
*Risk factors*				
History of bariatric surgery	0.44 (0.14, 1.32)	0.142	0.09 (0.01, 0.80)	0.031
Diabetes mellitus	3.99 (0.82, 19.34)	0.086	66.01 (6.61, 435.47)	<0.001
Obese	1.38 (0.40, 4.78)	0.615	0.78 (0.12, 4.94)	0.795

*Compared to pre-surgery. **Model 1: Odds ratio of upper tertile HOMA-IR; Model 2: Odds ratio of severely abnormal dwAG (dwAG3). HOMA-IR: Homeostasis model assessment-estimated insulin resistance; dwAG: Doi's weighted average glucose; SSFR: Surgical subcutaneous fat removal.

The odds of having upper tertile HOMA-IR (independent of the T2D status, BMI, and history of obesity surgery) was 30% higher (OR 1.30; *p* ═ 0.688) in the first week after SSFR but had dropped 78% below base value (OR 0.22; *p* ═ 0.042) by 6 weeks after SSFR (Table 2). The interpretation of the latter is that at 1 week after surgery the estimated OR suggested some worsening of HOMA-IR due to postoperative inflammatory status [26] but with little evidence against the null hypothesis at this sample size (*p* ═ 0.688). However, at 6 weeks, there was a clinically and statistically significant drop in HOMA-IR and the odds of upper tertile HOMA-IR dropped almost five-folds over the baseline.

On the contrary, those with a history of obesity surgery (irrespective of SSFR, BMI, and T2D status) had a 56% decrease in odds of upper tertile HOMA-IR (OR 0.44) compared to those without prior obesity surgery, but this time with some evidence against the null hypothesis at this sample size (*p* ═ 0.142). Diabetic status showed a four-folds higher odds of having upper tertile HOMA-IR (OR 3.99; *p* ═ 0.086). However, BMI had a weak independent correlation with insulin resistance status (OR 1.38; *p* ═ 0.615). This model showed the goodness of link (linktest in Stata) and goodness of fit (area under ROC curve= 0.709).

### Model 2 (dwAG3): Predictors of abnormal glucose excursion

The risk of having a severely abnormal glucose excursion on the GTT (defined as dwAG3) was assessed in relation to SSFR, history of bariatric surgery, as well as diabetic and obesity status independently (Table 2). The median dwAG in this severely abnormal group across all time points was 9.51 (IQR 9.15–11.93).

The odds of having severely abnormal dwAG (independent of the T2D status, BMI, and history of obesity surgery) was two-fold higher (OR 2.2; *p* ═ 0.256) in the first week after SSFR but had returned to the base value (OR 1.05; *p* ═ 0.956) by 6 weeks after SSFR. The interpretation of the latter is that at 1 week after surgery the estimated OR suggested some worsening due to postoperative inflammatory status [26] but there was weak evidence (*p* ═ 0.256) against the null hypothesis at this sample size.

On the contrary, those with prior obesity surgery had an almost ten-fold decrease in odds of a severely abnormal dwAG status (OR 0.09; *p* ═ 0.031) compared to those without prior obesity surgery (irrespective of SSFR, obesity, and T2D status).

Diabetic status as expected showed an extremely high odds of having severely abnormal dwAG (OR 66.01; *p* ═ 0.001). However, obesity status showed weak association with the risk of having a severely abnormal glucose excursion on the GTT (OR 0.78; *p* ═ 0.795) suggesting that abdominal fat mass and associated adipose fat dysfunction may be stronger predictors of insulin resistance status compared to total fat mass [27]. This model showed goodness of link (linktest in Stata) and goodness of fit (area under ROC curve = 0.764).

### The impact of prior bariatric surgery on glucose homeostasis changes after SSFR

**Figure 1.**
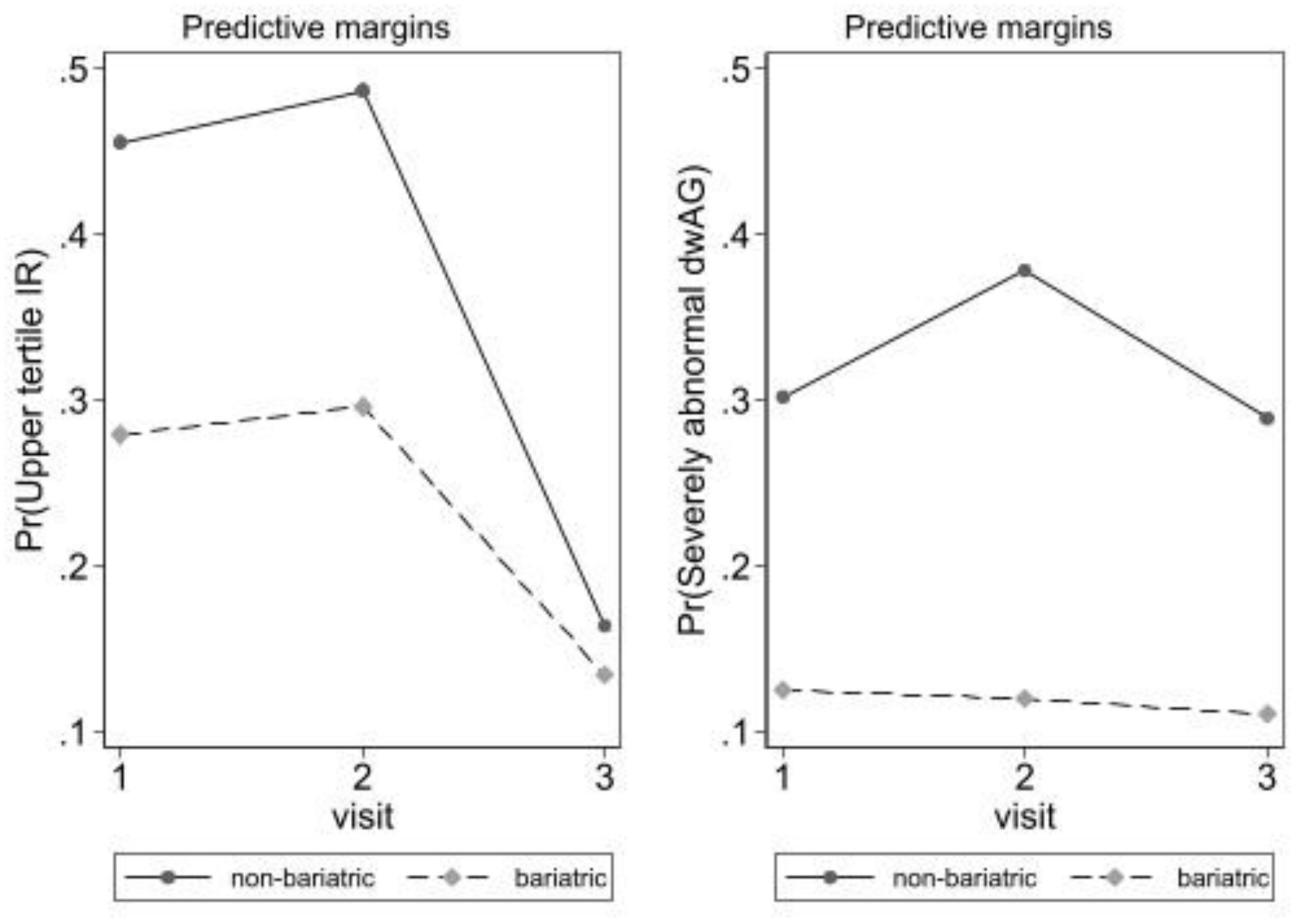
**Predictive margins after logistic regression in Table 2.** The left panel depicts insulin resistance (model 1; HOMA-IR) and right panel depicts glucose excursion under the GTT (model 2; dwAG). HOMA-IR: Homeostasis model assessment-estimated insulin resistance; dwAG: Doi's weighted average glucose; GTT: Glucose tolerance test.

The impact of prior bariatric surgery on changes in glucose homeostasis (both insulin resistance and glucose excursion under the GTT) after SSFR was examined using predictive margins after logistic regression from models 1 and 2. Figure 1 depicts the proportions under the models described in previous sections. This analysis aims to compare the changes in proportions of patients with either upper tertile insulin resistance or severely abnormal (dwAG3) glucose excursion under the GTT in those with the history of bariatric surgery vs bariatric surgery naïve participants. The left panel depicts results for upper tertile insulin resistance (model 1) and the right panel depicts results for dwAG3 glucose excursion under the GTT (model 2).

In the left panel (Figure 1), there is an increase in proportions with upper tertile insulin resistance by visit 2 and this is seen in both those with and without bariatric surgery history. Marked improvement then follows in visit 3 (again in both groups with and without history of bariatric surgery) suggesting that insulin sensitivity has improved markedly by 6 weeks (more so in bariatric surgery naïve participants). This also correlates with the previous finding above, where SSFR resulted in a transient worsening in insulin resistance at visit 2 (1 week after surgery) possibly due to the postoperative inflammatory status [26], followed by significant improvement at visit 3 (6 weeks after surgery).

The right panel in Figure 1 depicts the proportions in relation to severely abnormal glucose excursion (dwAG3) and here the picture is different. Those with a history of bariatric surgery have no real change in probability of this degree of glucose excursion over time while those without a prior history of bariatric surgery demonstrate a rise in the proportion with severely abnormal glucose excursion by visit 2 (which parallels the increase in HOMA-IR) and then returns to baseline by visit 3.

In both the left and right panels, those with a history of bariatric surgery have both lower proportions with gross insulin resistance as well as with severely abnormal glucose excursion at all time points. It is clear that the main impact of SSFR is on insulin resistance (HOMA-IR) in all subjects, but that the glucose excursion effect is markedly attenuated in those with a history of bariatric surgery.

These results clearly suggest that SSFR improves insulin sensitivity in those with or without bariatric surgery, but only impacts glucose excursion under the GTT in bariatric surgery naïve participants, suggesting that bariatric surgery results in sustained improvements in this area possibly related to better pancreatic β-cell function (and less so in terms of HOMA-IR) [28].

## Discussion

Obesity surgery is an efficient treatment for obesity and related metabolic diseases [29]. Because of the rapid and massive weight loss following the surgery, many patients tend to require body contouring plastic surgery to remove redundant abdominal skin and excess subcutaneous abdominal fat for aesthetic purposes. The precise mechanisms by which obesity surgery affords the protections and the consequences of surgical (and non-surgical) fat removal on human metabolism are not fully clear yet [5–7]. This study answered a few of the pertinent questions through examination of the early postoperative changes in glucose homeostasis after SSFR at three time points. A clear protective effect of prior obesity surgery on glucose excursion during the GTT was demonstrated using a novel index, the dwAG. This effect was found to be independent of time post SSFR, BMI, and diabetic status. Abnormal glucose excursion has been associated with different metabolic risk profiles and increased future risk of T2D [30]. Therefore, our results suggest that obesity surgery offers this protection, independent of BMI. The mechanism underpinning this protection on abnormal glucose excursion seems to work through both effects on insulin resistance as well as pancreatic β cell function because the OGTT combines both insulin resistance and the β cell function status [31]. The implication is that glucose excursion under the OGTT curve provides a predictive test for the future development of T2D, independent of BMI. The latter is related to the overall shape of the glucose excursion curve and thus the slower the glucose curve returns to the fasting glucose level, the worse the metabolic profile with greater insulin resistance and/or worse pancreatic β cell function, and higher risk of future development of T2D [30, 32].

Insulin resistance, which is defined as a suboptimal response to normal blood levels of insulin, is what links overweight and obesity to worsening pancreatic β cell function, T2D and its associated metabolic consequences such as cardiovascular diseases. In this study, subjects with a history of obesity surgery had a markedly lower glucose excursion even at visit 2 when HOMA-IR increased, strongly suggesting that the obesity surgery effect is mediated through sustained improvement in pancreatic β cell function. This is interesting because obesity surgery is known to improve glucose homeostasis before significant weight loss ensues [32, 33] and this also occurs with calorie restriction [34]. The mechanisms by which pancreatic cell health and function are improved remain unknown [35] though it has been suggested that gut hormones, especially glucagon-like peptide-1 [36], may modulate this effect. Better understanding of what happens in the aftermath of obesity surgery will provide novel insights into our understanding of the management of chronic metabolic sequalae of obesity, especially T2D.

The removal of about 2–3 kg of ASF (through SSFR) was associated with a net benefit in terms of insulin resistance post SSFR as indicated in Table 2 and Figure 1 at 6 weeks. This improvement in insulin resistance may be linked to SSFR-associated changes in the secretion of certain adipokines, such as leptin and IL-6 [37, 38]. These two adipokines are secreted from subcutaneous fat stores rather than the visceral fat stores, due to their larger mass and higher secretion rate [38]. They both act centrally (in the hypothalamus) and peripherally in various tissues, such as adipocytes, pancreas, liver, and skeletal muscles [39], to promote insulin action and sensitivity, thereby maintaining glucose homeostasis. However, the impact of these primary adipokines may be influenced by other factors, particularly in cases of elevated leptin levels in obesity, and these additional factors may counteract the favorable effects of leptin [40, 41].

There is no doubt that leptin exerts an insulin-sensitizing effect since leptin administration exerts an insulin-sensitizing effect in those with low leptin states, including lipoatrophy states [43] and hypoleptinaemic states are also associated with insulin resistance [41, 43] which can be ameliorated by leptin treatment [43, 44]. Thus, a decrease in leptin levels is expected after SSFR, but the underlying mechanism for the paradoxical improvement in insulin sensitivity remains unknown. One explanation could be that leptin resistance is a consequence of deficiency of some other adipokine that is deficient in obesity and rises after SSFR. This would ease leptin from its resistant state, even as its own levels decrease. The mechanisms involved however need further investigation to establish a link with the two main adipokines—leptin and IL-6, which are the most abundant adipokines secreted from white adipose tissue [45].

## Conclusion

In conclusion, this study demonstrates an improvement in insulin resistance after SSFR, independent of BMI, diabetic status, or obesity surgery status. Furthermore, this study sheds new light on the possibility that the long-term impact of obesity surgery may primarily target improvement in pancreatic β-cell function, regardless of SSFR. However, the intricate interplay between SSFR and obesity surgery in obesity and T2D remains to be fully elucidated.

## Supplemental Data

**Figure S1.**
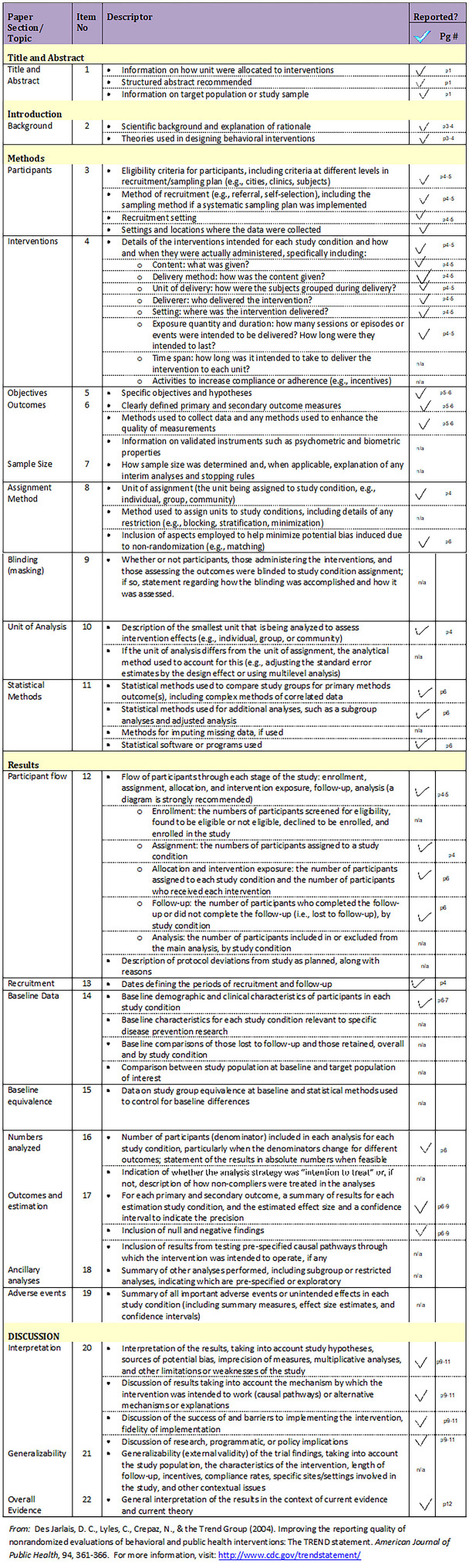
Trend statement checklist.
